# Treatment with the IDO inhibitor INCB024360 increased lysis of human tumor cell targets by peptide-specific CTL

**DOI:** 10.1186/2051-1426-3-S2-P275

**Published:** 2015-11-04

**Authors:** Caroline Jochems, Anna Kwilas, Young-Seung Kim, Martin W Brechbiel, Massimo Fantini, Simon Metenou, Romaine I Fernando, Peter S Kim, Sofia Gameiro, James Hodge, Robert Newton, Jeffrey Schlom, Kwong Y Tsang

**Affiliations:** 1 National Cancer Institute, National Institutes of Health, Bethesda, MD, USA; 2 Radiation Oncology Branch, NCI, NIH, Bethesda, MD, USA; 3 NCI (National Cancer Institute), NIH, Bethesda, MD, USA; 4 Laboratory of Tumor Immunology and Biology, Center for Cancer Research, National Cancer Institute, National Institutes of Health, Bethesda, MD, USA; 5 Incyte Corporation, Wilmington, DE, USA

## 

We have investigated the *in vitro* effects of INCB024360 on dendritic cell maturation and activation of antigen-specific T cells. INCB024360 is a novel inhibitor of indoleamine-2, 3-dioxygenase (IDO), and is currently in several ongoing clinical trials. INCB024360 effectively suppresses systemic tryptophan catabolism and tumor growth.

Human dendritic cells (DC) were generated from PBMCs from healthy donors. The tryptophan (Trp) and kynurenine (Kyn) concentrations in supernatants, and the Trp/Kyn ratio, were measured by HPLC. The Trp level was 75.8% in supernatants of immature DCs with little breakdown to Kyn (Kyn/Trp ratio of 0.32). In contrast, in supernatants of matured DCs, the Kyn/Trp ratio was 5.9 for IFNγ matured DCs, and 9.2 for IFNγ/LPS matured DCs. Treatment with INCB024360 resulted in almost no breakdown of tryptophan. The expression levels of several DC activation markers did not change after treatment with INCB024360.

We then compared the efficacy of antigen presentation by DCs treated with and without the inhibitor. MUC1-C peptide specific CTL were derived from a prostate cancer patient. DCs pulsed with peptide and treated with INCB024360 stimulated the CTL to produce more IFNγ and other type I cytokines than untreated DCs.

A MUC1-C-specific, HLA-A24^+^ was stimulated using its specific MUC1 peptide and DCs treated with INCB024360. The T cells were used in a CTL assay using PC3 (human prostate carcinoma, MUC1^+^, HLA-A24^+^) as a target and ASPC-1 (human pancreatic carcinoma, MUC1^+^, HLA-A24^NEG^) as a negative control. Pre-treating the DCs with INCB024360 resulted in increased tumor cell lysis. An additional T cell line, specific for brachyury peptide and HLA-A2, was derived from another prostate cancer patient, and after stimulation with INCB treated DCs the lysis of the human breast cancer cell line increased.

These results thus demonstrated that INCB024360 treatment of DCs resulted in both increased cytokine production and increased tumor cell lysis by antigen-specific CD8^+^ T cell lines derived from cancer patients. This suggests that INCB024360 could potentially be effectively combined with other immune modulating therapies. Murine anti-tumor studies of INCB024360 alone or in combination with vaccine are ongoing.

